# Can aura migraine be elicited by isolated pulmonary arteriovenous fistula?—A case report

**DOI:** 10.3389/fneur.2022.1079959

**Published:** 2022-12-15

**Authors:** Eustaquio Maria Onorato, Josephal Salvia, Mariano Becchina, Tommaso Cipolla, Gian Paolo Anzola

**Affiliations:** ^1^R.C.C.S. Ospedale Galeazzi-Sant'Ambrogio GSD, Milan, Italy; ^2^Cardiology Department, Istituto Fondazione G. Giglio di Cefalù, Palermo, Italy; ^3^Neurology Clinic and Rehabilitation Department, Casa di Cura Villa Barbarano, Brescia, Italy

**Keywords:** pulmonary arteriovenous malformations, migraine aura, hereditary hemorrhagic telangiectasia, right-to-left shunt, patent foramen ovale, Transcranial Doppler

## Abstract

A pulmonary arteriovenous fistula (PAVM) is an abnormal blood vessel that creates a direct connection between a pulmonary artery and its tributary vein bypassing capillary filter, establishing as a consequence of a low-resistance right-to-left shunting (RLS). The vast majority of PAVMs are congenital appearing more often in females than in males. A great number of patients with PAVMs is suffering concurrently from hereditary hemorrhagic telangiectasia (HHT) whose incidence is around 1 in 5,000. Very few cases of acquired PAVMs have been described in the literature. Paradoxical embolism through PAVMs can cause systemic desaturation, cyanosis, and serious cerebrovascular ischemic events (transient ischemic attacks, strokes, and intracranial abscess), even when the abnormal blood vessel is small (diameter <3 mm). Notably, it has been reported a high prevalence of aura migraine (MHA) symptoms in patients with PAVMs and concomitant HHT. We described in this study the case of a young aura migraineur female patient without HHT in whom isolated PAVM below the detection limit of pulmonary angiography and chest computed tomography angiography (CTA) has been documented by contrast Transthoracic and Transesophageal Echocardiography (cTTE/TEE) showing a delayed (>17 s) RLS coming from left pulmonary veins to left atrium while a patent foramen ovale (PFO), small atrial septal defects or septum primum fenestration could not be detected despite several attempts. Contrast Transcranial Doppler (cTCD) confirmed a delayed (>16 s) RLS with two short “shower” patterns corroborating the diagnosis of an extra-cardiac RLS. During the right heart catheterization and pulmonary angiography, it was impossible to cross the interatrial septum with a multipurpose catheter. The patient was finally discharged with off-label thienopyridine agents (clopidogrel 75 mg die) in terms of primary prophylaxis for paradoxical right-to-left embolization of thrombotic material ultimately. Aura migraine symptoms were nearly abolished by P2Y12 platelet inhibition, suggesting a platelet-based mechanism. During 2 years of clinical follow-up on thienopyridine therapy, the resolution of aura migraine episodes was definitively accomplished with significant improvement in her quality of life.

## Introduction

A pulmonary arteriovenous fistula (PAVM) is defined as a congenital abnormal communication between the pulmonary artery and its tributary vein creating a low-resistance, high-flow right-to-left shunting (RLS) bypassing the capillary bed (1, 2). The vast majority of PAVMs are congenital. Isolated PAVMs are uncommon. Their incidence has been reported as high as 1 in 2,600 (3) and occur in about one-third of all patients with hereditary hemorrhagic telangiectasia (HHT) also known as Rendu–Osler–Weber disease, an autosomal dominant disorder caused by a mutation of endoglin gene (HHT type 1) or activin receptor-like kinase 1 (HHT type 2) and characterized by abnormalities of vascular structures involving multiple organs (4, 5). A third gene, the one that codes for the transcription factor SMAD4, is responsible for the HHT associated with Juvenile Polyposis (6, 7). PAVMs may be present with a variety of clinical manifestations such as dyspnea, hypoxemia, chest pain, and pulmonary hypertension. Cryptogenic strokes from isolated PAVMs have been described but they are rare (8, 9). Migraines linked to intrapulmonary RLS have been described (10–12) and are sometimes the presenting clinical manifestation of PAVMs. We reported such a case of isolated intrapulmonary RLS below the detection limit of pulmonary angiography and computed tomography angiography in a patient presenting with severe aura migraine successfully treated with P2Y12 platelet inhibition.

## Case

A 38-year-old woman without cardiovascular risk factors was suffering since the age of 14 from endometriosis refractory to non-steroidal anti-inflammatory drugs. At the age of 25, progesterone replacement therapy (PRT) was prescribed with a significant decrease in pain and cramps around menstruations, but worsening episodes of migraine with aura concomitantly occurred. She reported no neurologic symptoms. On neurological examination, strength, skin sensation, visual field, reflexes, and coordination were normal.

She was suffering since childhood from prodromal symptoms (sensitivity to light) preceding recurrent attacks of unilateral reversible visual aura and sensory symptoms (numbness) that were usually followed by intensive headache lasting up to 24 h occurring two times a week with nausea often followed by emesis with aggravation of the headaches, meeting International Classification of Headache Disorders−3rd edition for migraine aura. Tryptans did not ameliorate her headaches. T1-, T2-, T2-FLAIR, and diffusion-weighted brain magnetic resonance imaging (MRI) showed scanty focal bilateral supratentorial white matter hyperintensities (WMHs).

Pregnancy was planned and HRT was suspended. After an uneventful delivery, HRT was re-started and a severe migraine aura occurred thereafter.

## Diagnostic assessment

**Figure 1:**
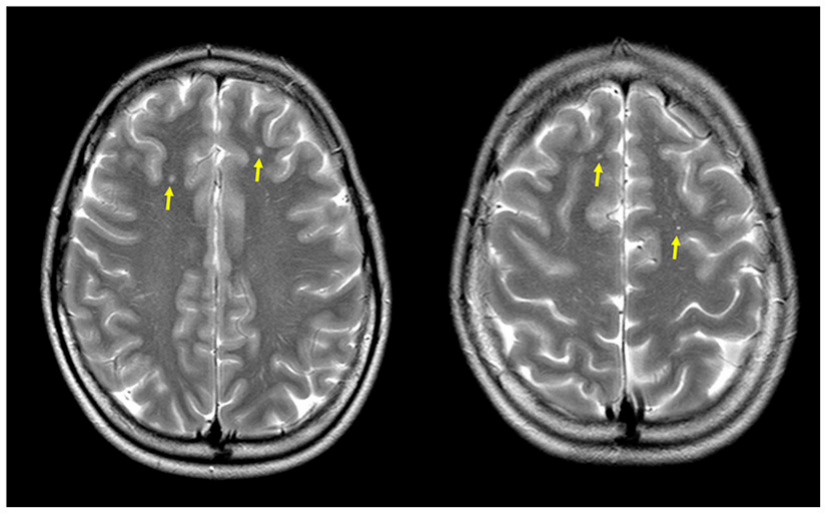
Magnetic resonance (MR) axial T2-weighted-FLAIR images showing multiple focal bilateral supratentorial white matter hyperintensities (yellow arrows).

On control MRI 1 year later, WMHs of the same size and number were identified (Figure 1) as it has been reported in shunt-associated migraine (SAM) patients (13).

**Figure 2:**
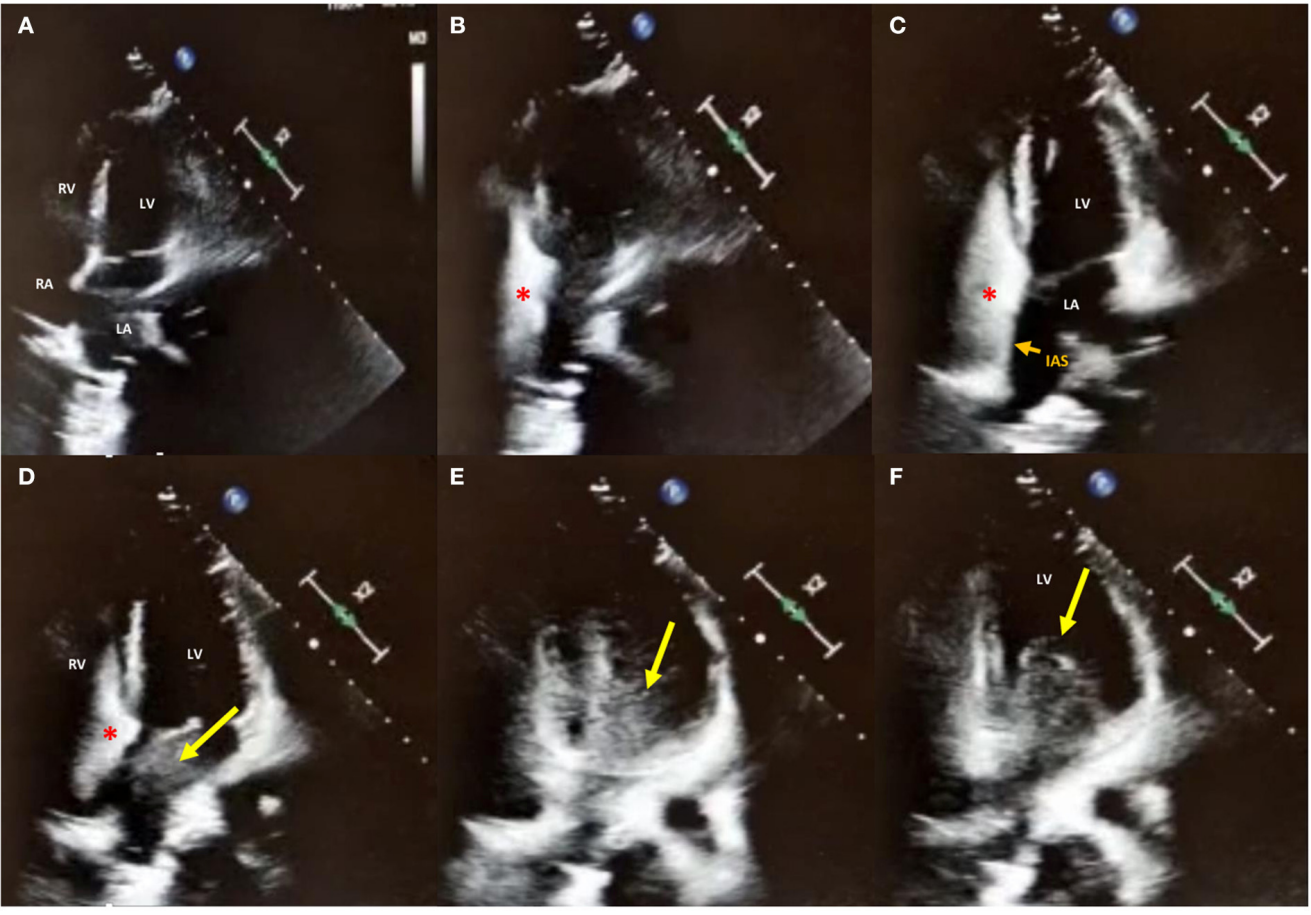
2D contrast-Transthoracic Echocardiography in apical 4-chambers view in basal conditions. **(A–C)** Agitated saline injection (red asterisk) from the right antecubital vein showed no right-to-left shunt (RLS) across the interatrial septum (IAS; small orange arrow); **(D–F)** While still no bubbles were crossing the interatrial septum, a delayed (17 s) RLS coming from left pulmonary veins to LA and LV through the mitral valve is shown, confirming the diagnosis of extra-cardiac RLS. LA, left atrium; RA, right atrium; LV, left ventricle; RV, right ventricle.

**Figure 3:**
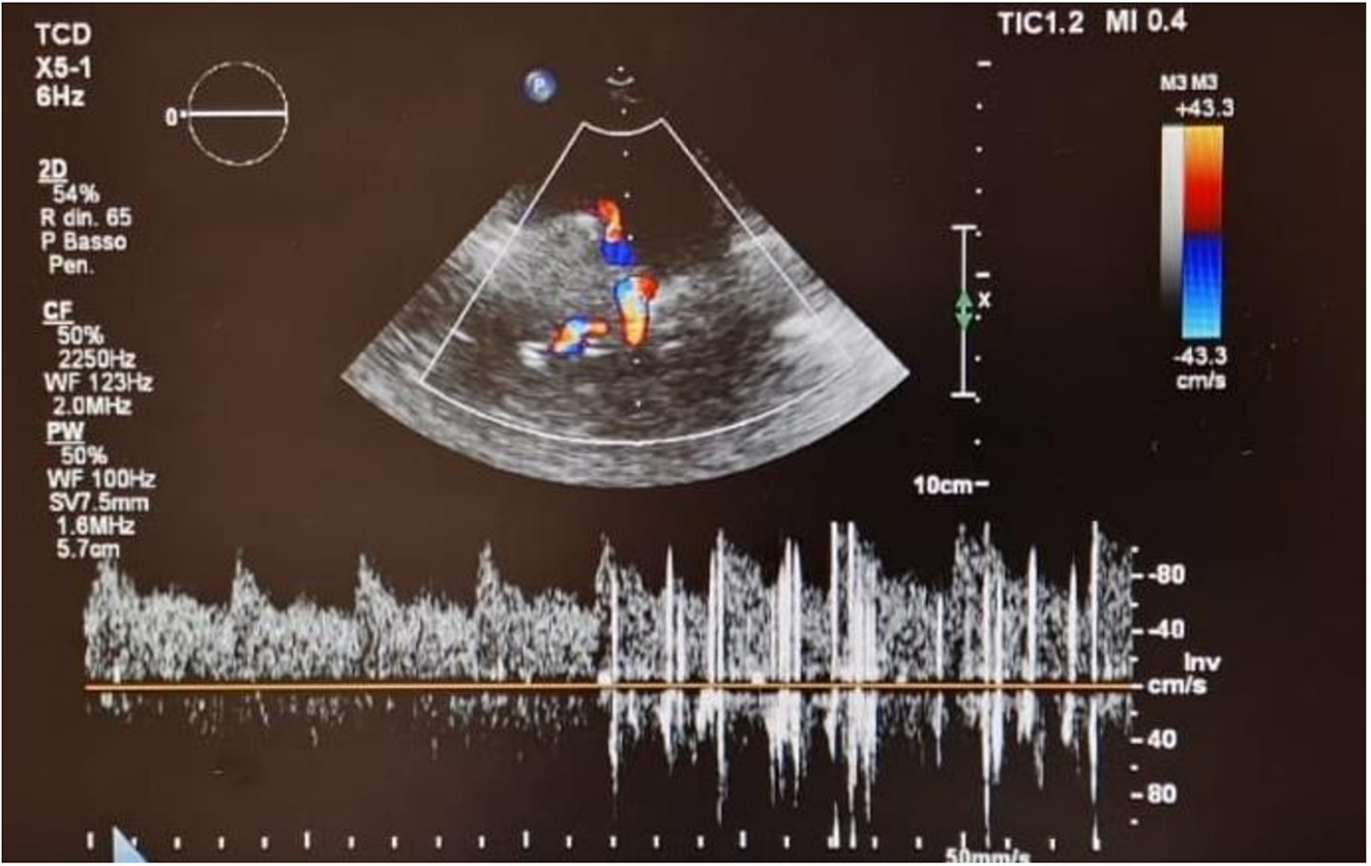
Contrast Transcranial Doppler in basal conditions showing high-intensity transient signals passing through the middle cerebral artery with a delayed (17 s) “shower” pattern.

Electrocardiogram showed persistent sinus rhythm and incomplete right bundle branch block. Chest X-ray, computed tomography angiography (CTA), and 2D TTE/TEE color Doppler were performed resulting within normal limits. Contrast TTE/TEE using agitated saline solution showed no RLS across the interatrial septum (Figure 2, Supplementary Videos 1, 2) but conversely demonstrated bubbles arriving from left pulmonary veins to the left atrium and left ventricle *via* a pulmonary arteriovenous fistula (Supplementary Figure 1) in basal conditions. Contrast-enhanced Transcranial Doppler (cTCD) raised the suspicion of an extracardiac RLS by showing in basal conditions high-intensity transient signals passing through the middle cerebral artery with a delayed (17 s) “shower” pattern (Figure 3, Supplementary Video 2). Of note, aura migraine symptoms occurred 4 min after agitated saline injection (“bubble migraine positive” patient) (14). According to the protocol of the Venice 1999 Consensus conference, the bubble count was performed twice, during normal breathing and after Valsalva strain (15). It is worth to mention that no evidence or family history of HHT was documented in her past medical history.

**Figure 4:**
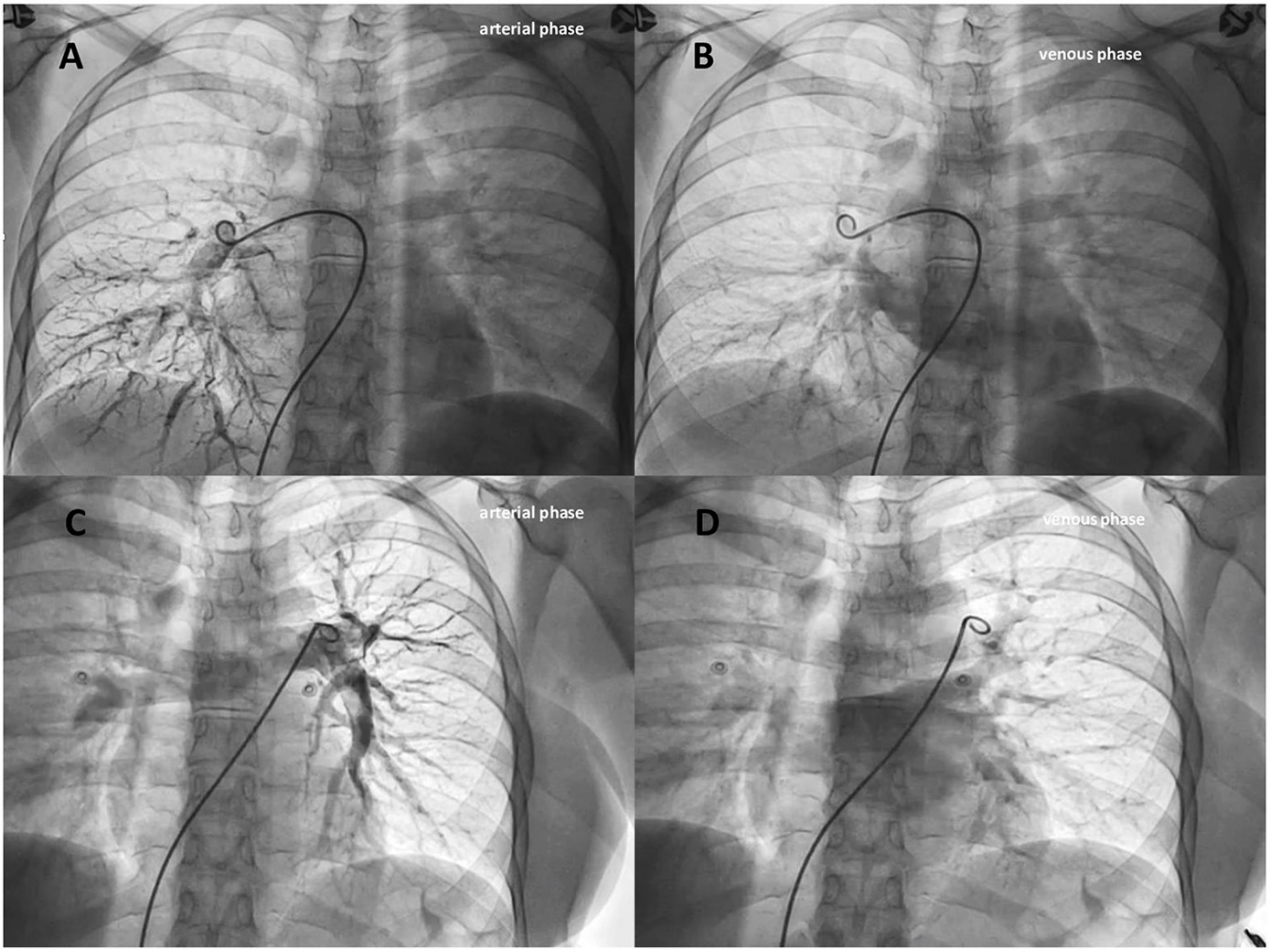
Selective right **(A,B)** and left **(C,D)** pulmonary angiograms (arterial and venous phases) didn't show abnormalities of the pulmonary vasculature or arteriovenous shunts.

The decision was made by our multidisciplinary heart and brain team to offer a clinical re-evaluation in order to stratify the risk of paradoxical embolism and consequently to perform possibly a catheter-based embolization using detachable coils or vascular plugs of the isolated intrapulmonary RLS. Written informed consent, after explanation, was obtained from the patient. During the hospital stay, she was monitored for vital parameters without any abnormalities except for a slight reduction in arterial oxygen saturation (SaO_2_) of 94%. Continuous ECG ruled out atrial fibrillation. Right heart catheterization to evaluate hemodynamic parameters and selective pulmonary angiograms were performed in order to definitively check for an isolated PAVM and to search for accompanying malformations. Right atrial pressure, pulmonary artery pressure, pulmonary vascular resistance, and pulmonary to systemic flow ratio (QP/QS) were within normal limits. It has been impossible to cross the interatrial septum with a multipurpose catheter despite multiple attempts. Oddly, selective right and left pulmonary arteriograms did not show abnormalities of the pulmonary vasculature or arteriovenous shunts (Figure 4, Supplementary Video 3). Brachiocephalic vein angiogram ruled out the presence of persistent left superior vena cava draining into the left atrium, another possible rare cause of RLS and paradoxical embolism (Supplementary Video 4) (16).

The Heart and Brain team then decided to discharge the patient on off-label thienopyridine agents (clopidogrel 75 mg die) in terms of primary prophylaxis for paradoxical embolization of thrombotic material that could bypass the pulmonary capillary filter. Surprisingly, a substantial reduction in aura migraine symptoms in our patient was achieved using P2Y12 platelet inhibition, suggesting a platelet-based trigger. HRT was advantageously resumed without migraine attacks. During 2 years of follow-up on thienopyridine therapy, the resolution of migraine aura episodes was accomplished.

## Discussion

Increased prevalence of migraines has been described in patients with HHT, the most common cause of PAVMs (1–4) as well in patients with intrapulmonary RLS shunts (11, 17, 18). Thenganatt et al. pointed out that PAVMs were significantly associated with migraine (OR = 2.4, 95% CI = 1.1–5.5, *p* = 0.04), after adjustment for age and sex, using logistic regression (10).

Our patient had no personal evidence or family history of HHT and was diagnosed with isolated congenital PAVM. The frequency of migraine headaches in patients with isolated PAVM is unknown. A causal relationship between the presence of an RLS and migraines has been suggested but remains unproven until now. Several hypotheses have been suggested. Firstly, being HHT and some subtypes of migraines autosomal-dominant disorders, it might be that a particular genetic substrate determining intrapulmonary RLS in patients with HHT may also activate migraines (19). Secondly, trigger substances such as micro embolic particles or vasoactive chemicals (serotonin) might enter directly the systemic circulation through the RLS instead of being inactivated in the pulmonary capillary bed. These vasoactive substances might induce instability or increased excitability of central nervous system, causing aura migraine attacks (20). Thirdly, micro embolic air load could act as a trigger of migraine attack provoking a sudden decrease of oxygen saturation in cerebral circulation triggering cortical spreading depression and as a result migraine attack (14).

A further mechanism strictly interacting with those mentioned could be identified in cerebral hemodynamics of MHA patients. Evidence support the role of increased cerebral vasomotor reactivity in MHA patients with large right-to-left shunt and an altered cerebral autoregulation in aura migraineurs (21, 22).

Furthermore, a high prevalence of migraines (up to 59%) has been shown in patients who were admitted for transcatheter closure of large PAVMs (9, 23). However, the effects of embolization on the prevalence of migraines have not been confirmed. Only one study has reported a significant reduction in migraine episodes after embolization of PAVMs (24).

Based on the Second International Guidelines for the diagnosis and management of HHT (25), confirmation of intrapulmonary shunts should be performed by CTA with thin-cut reconstructions, in order to evaluate the necessity for endovascular embolization. Nevertheless, additional chest CTA with thin-cut reconstructions should not be performed in cases with only an isolated intrapulmonary RLS documented by cTTE/TEE and cTCD, as these shunts may be too small for subsequent transcatheter embolization procedure.

Of note, the availability of simple diagnostic techniques such as cTCD and cTTE/TEE to assess RLS undoubtedly contributed a great deal of knowledge and understanding of this rare clinical entity.

Historically, progressive expansion of the PAVMs and paradoxical embolism are the main two indications for surgical ligation and resection (26). Technological improvements have made endovascular embolization the treatment of choice for most patients (27, 28), and this represents a clear advantage in comparison to surgical ligation and resection. The exact number, size, and location of PAVMs confirmed by pulmonary angiography can be very helpful guidance to tailor the percutaneous treatment to each patient's clinical condition and anatomical characteristics. Noteworthy, while transcatheter embolization is considered first-choice treatment for PAVMs, a significant amount of patients successfully treated may experience recurrences in the follow-up.

Based on observational series, Sommer et al. (29) demonstrated that in the PFO-MHA population treatment with thienopyridines (platelet P2Y12 receptor inhibitors) substantially reduced or eliminated migraine symptoms in up to two-thirds of patients. Platelet aggregation or platelet activation byproducts from the venous circulation, rather than being filtered in the lungs, might cross the PFO or an extra-cardiac source like an intrapulmonary fistula as in our case to reach the brain in exceeding levels, triggering the MHA. That assumption was substantiated by the observation that the MHA response to P2Y12 platelet inhibition correlated nearly perfectly with the MHA response to subsequent PFO closure.

More recently, laboratory analysis of platelet function in patients with PFO and MHA demonstrated that there is a hyperactive platelet state resulting in an increased endogenous thrombin potential and an altered oxidative stress status documented by increased platelet reactive oxygen species production and blood oxidized glutathione/reduced glutathione ratio. Concomitantly, the markers of platelet hyperactivity returned to control levels and coincided with MHA cessation with the administration of clopidogrel or with subsequent catheter-based PFO closure (30). Accordingly, with Sommer and more recent literature, this platelet prothrombotic phenotype was better controlled by P2Y12 antagonist while aspirin had little effect on it (31).

In summary, we report a case of a patient with isolated intrapulmonary RLS below the detection limit of pulmonary angiography and CTA whose aura migraine symptoms resolved following P2Y12 platelet inhibition with a considerable improvement in her quality of life. Further studies are needed to determine the association between intrapulmonary RLS and migraine and whether MHA symptoms can be elicited by isolated pulmonary arteriovenous fistula.

## Data availability statement

The raw data supporting the conclusions of this article will be made available by the authors, without undue reservation.

## Ethics statement

Written informed consent was obtained from the patient for the publication of any potentially identifiable images or data included in this article.

## Author contributions

EO, JS, and TC contributed to the conception and design of the study. EO, JS, MB, and GA contributed to drafting the text or preparing the figures. All authors contributed to the article and approved the submitted version.

## Supplementary material

Supplementary Figure 1 2D contrast-Transthoracic Echocardiography (cTTE) showing agitated saline solution (yellow arrow) coming from left superior pulmonary vein draining into the left atrium. LAA, left atrial appendage; LSPV, left superior pulmonary vein.

Supplementary Video 1 2D contrast-Transthoracic Echocardiography (cTTE) after Valsalva maneuver showed a delayed (16 s) RLS coming from left superior pulmonary vein to LA (extra-cardiac RLS).

Supplementary Video 2 2D contrast-Transesophageal Echocardiography (cTEE) in basal conditions **(A)** and after Valsalva maneuver **(B)** showing non bubbles crossing the interatrial septum.

Supplementary Video 3 Selective right **(A)** and left **(B)** pulmonary angiograms in the anteroposterior projection showing no abnormalities of the pulmonary vasculature or intrapulmonary arteriovenous malformations.

Supplementary Video 4 Brachiocephalic vein angiogram showed a remnant of the persistent left superior vena cava but ruled out its draining into the left atrium.
