# Supplementary figures and images for: Can aura migraine be elicited by isolated pulmonary arteriovenous fistula?—A case report
Source: Front Neurol. 2022 Dec 15;13:1079959. doi: 10.3389/fneur.2022.1079959 (PMC9797860; doi:10.3389/fneur.2022.1079959)

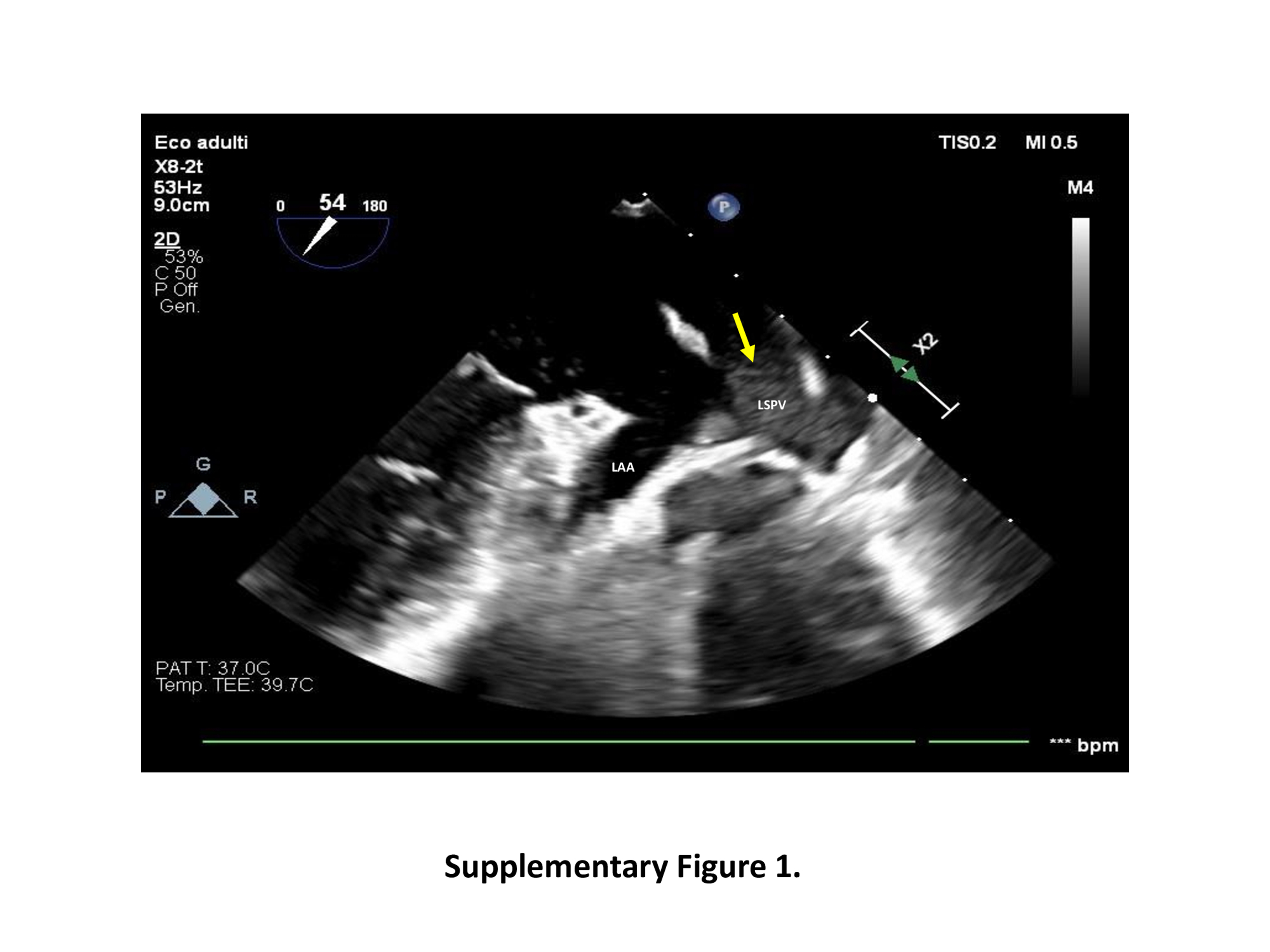

Supplement: Supplementary Figure 1 — 2D contrast-Transthoracic Echocardiography (cTTE) showing agitated saline solution (yellow arrow) coming from left superior pulmonary vein draining into the left atrium. LAA, left atrial appendage; LSPV, left superior pulmonary vein. [file Image_1.JPEG]

## Slide 1
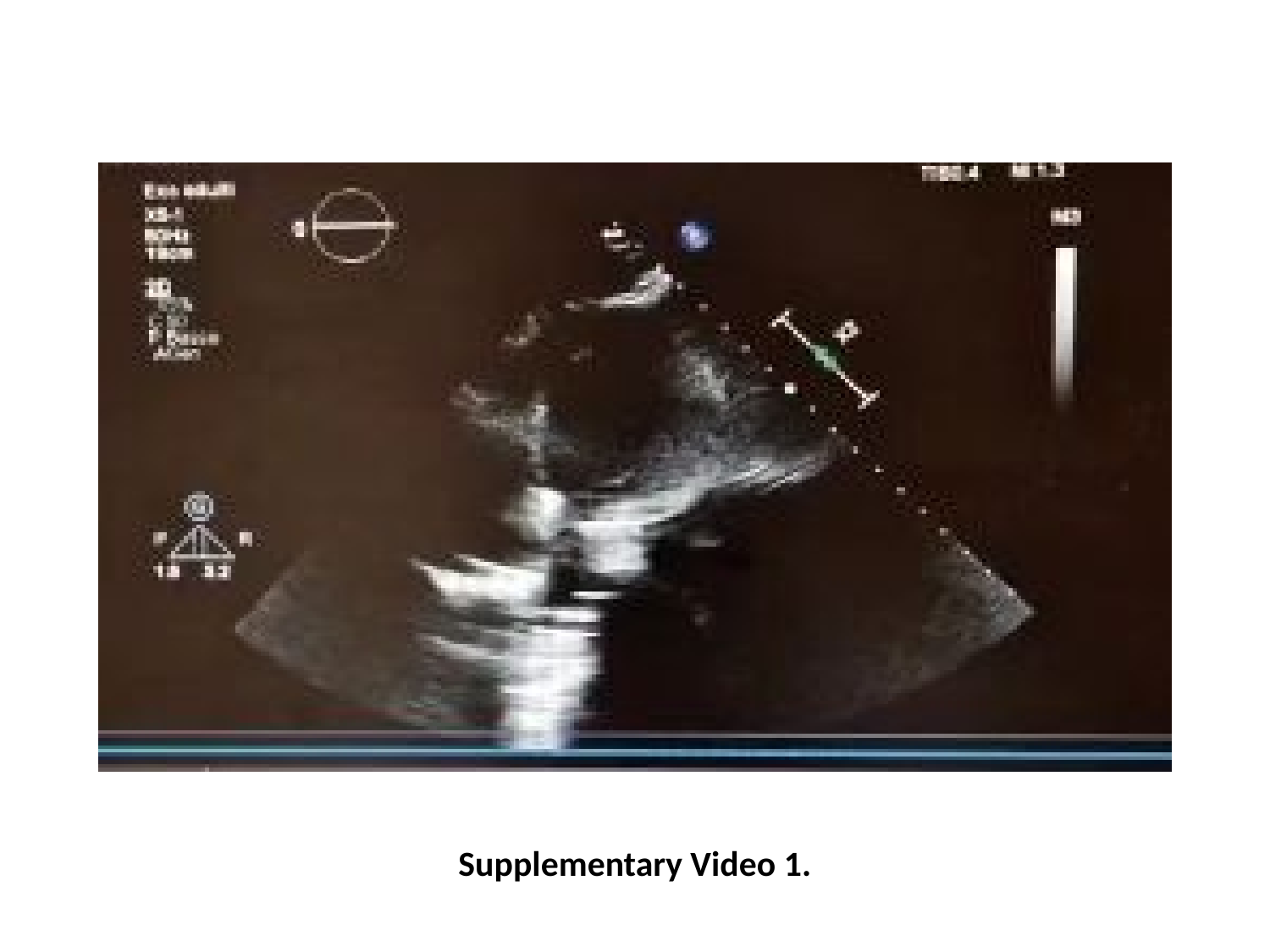

A
B
Supplementary Video 1.
LA

Supplement: Supplementary Video 1 — 2D contrast-Transthoracic Echocardiography (cTTE) after Valsalva maneuver showed a delayed (16 s) RLS coming from left superior pulmonary vein to LA (extra-cardiac RLS). [file Presentation_1.PPTX]

## Slide 1
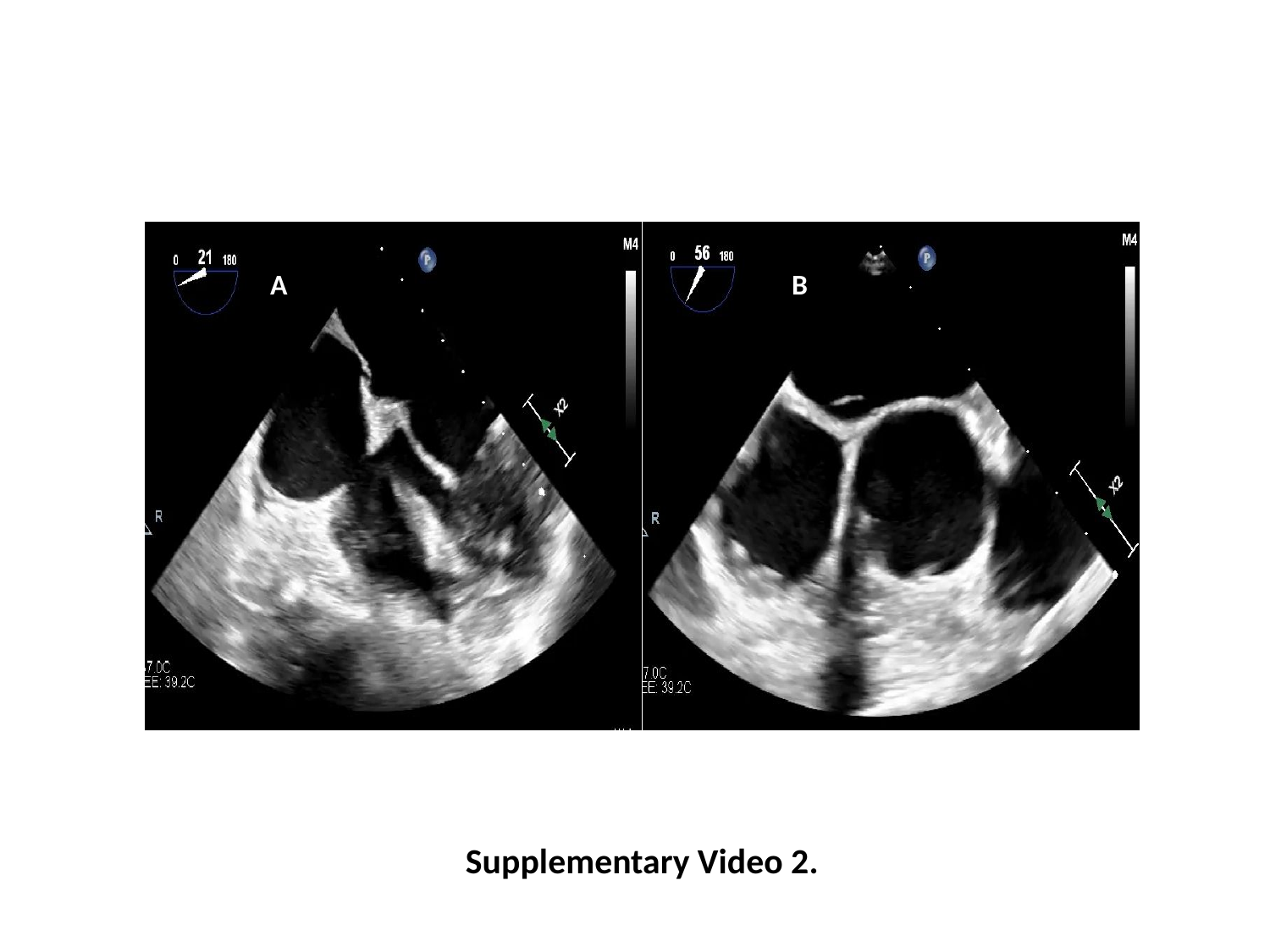

A
B
Supplementary Video 2.
LA

Supplement: Supplementary Video 2 — 2D contrast-Transesophageal Echocardiography (cTEE) in basal conditions (A) and after Valsalva maneuver (B) showing non bubbles crossing the interatrial septum. [file Presentation_2.PPTX]

## Slide 1
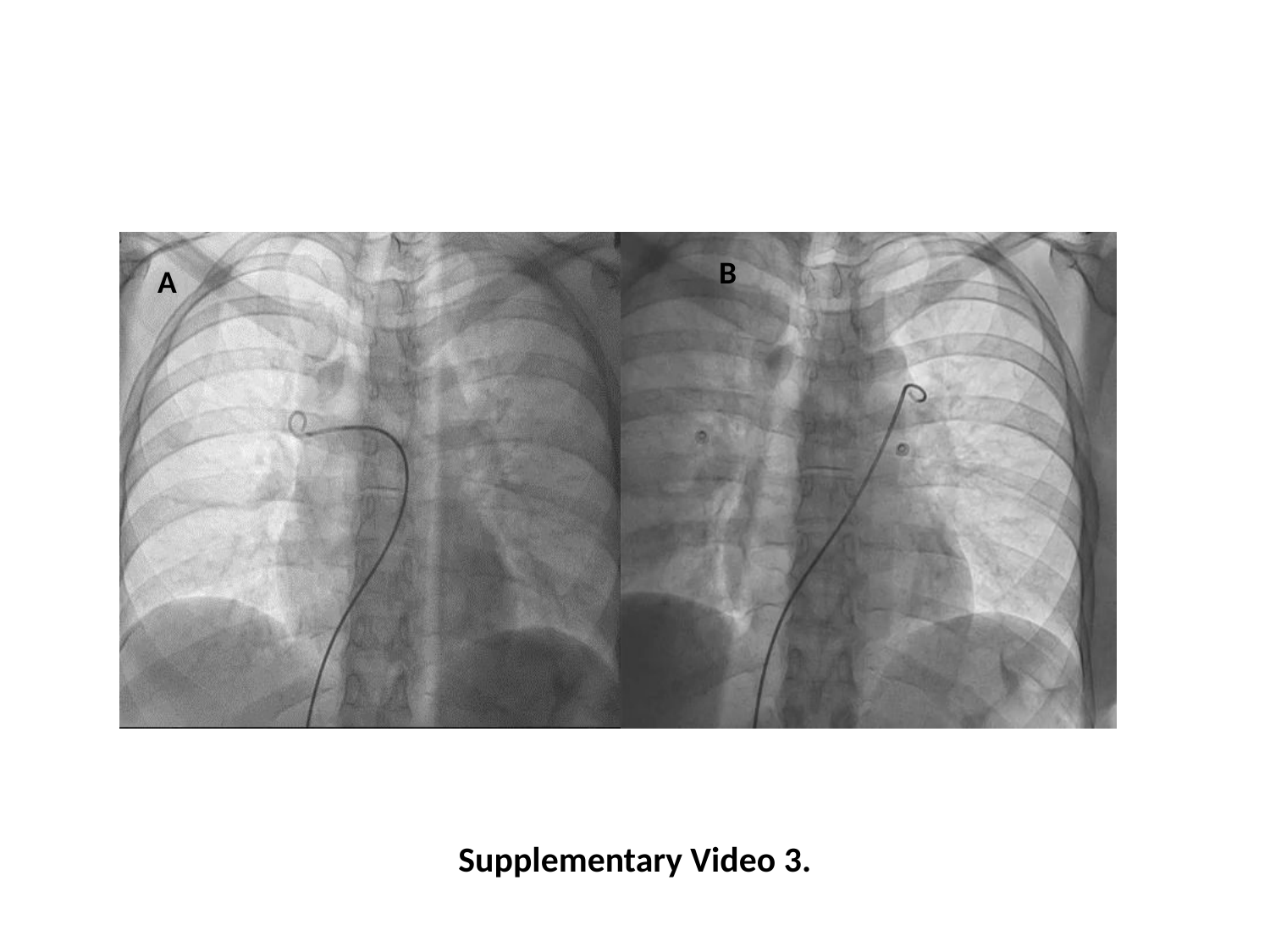

B
A
Supplementary Video 3.
LA

Supplement: Supplementary Video 3 — Selective right (A) and left (B) pulmonary angiograms in the anteroposterior projection showing no abnormalities of the pulmonary vasculature or intrapulmonary arteriovenous malformations. [file Presentation_3.PPTX]

## Slide 1
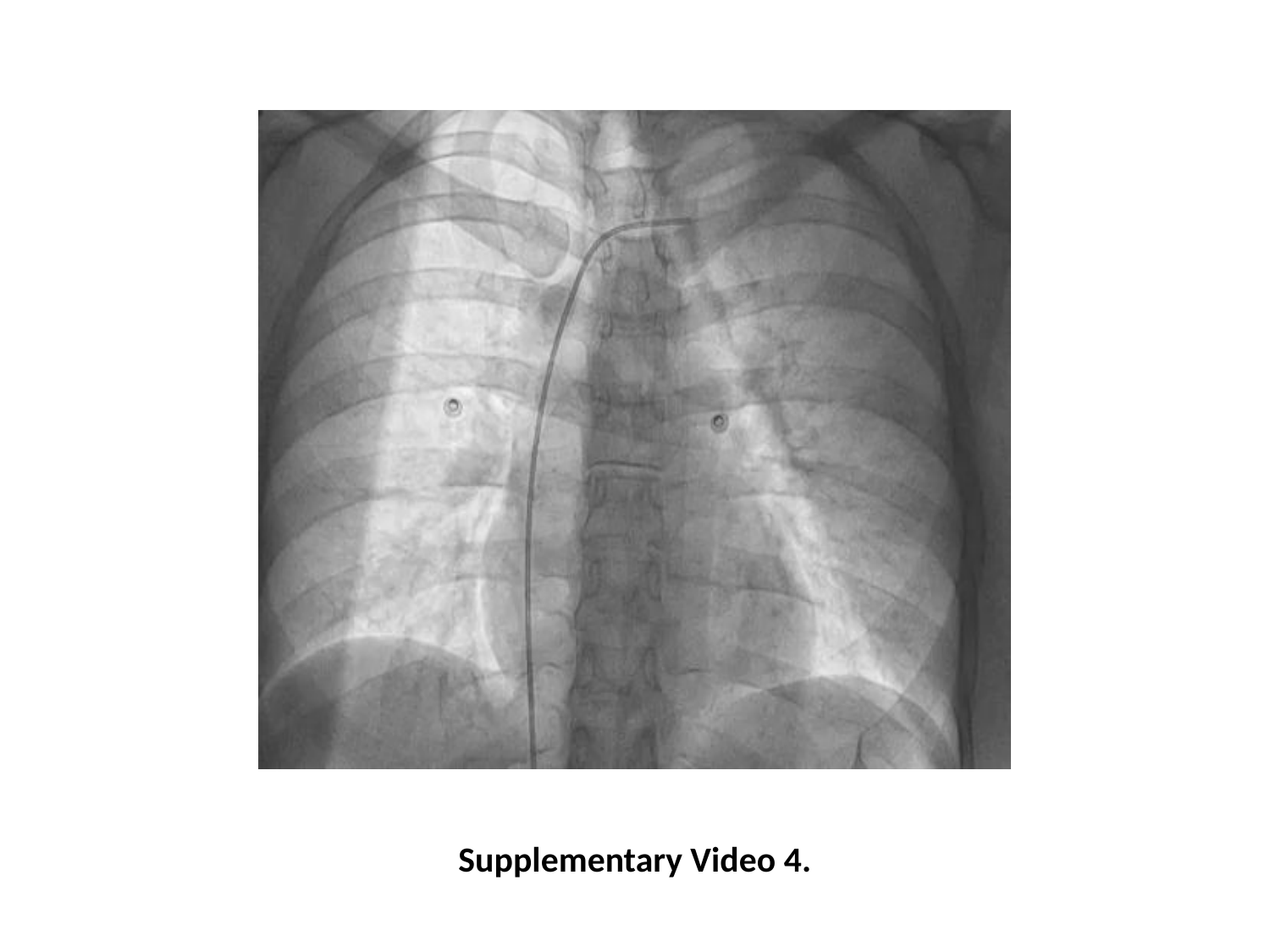

Supplementary Video 4.
LA

Supplement: Supplementary Video 4 — Brachiocephalic vein angiogram showed a remnant of the persistent left superior vena cava but ruled out its draining into the left atrium. [file Presentation_4.PPTX]
